# The role of patients’ stories in medicine: a systematic scoping review

**DOI:** 10.1186/s12904-023-01319-w

**Published:** 2023-12-12

**Authors:** Elaine Li Ying Quah, Keith Zi Yuan Chua, Casper Keegan Ronggui Lin, Andrew Vimal Vijayan, Nur Amira Binte Abdul Hamid, Jasmine Lerk Juan Owyong, Neeta Satku, Natalie Woong, Crystal Lim, Gillian Li Gek Phua, Eng Koon Ong, Warren Fong, Lalit Kumar Radha Krishna

**Affiliations:** 1 Yong Loo Lin School of Medicine, National University Singapore, Level 11 NUHS Tower Block, 1E Kent Ridge Road, Singapore, 119228 Singapore; 2 Division of Supportive and Palliative Care, National Cancer Centre Singapore, 30 Hospital Boulevard, Singapore, 168583 Singapore; 3 Centre for Biomedical Ethics, National University of Singapore, Blk MD11, 10 Medical Drive, #02-03, Singapore, 117597 Singapore; 4 Division of Outpatient Pharmacy, National Cancer Centre Singapore, 30 Hospital Boulevard, Singapore, 168583 Singapore; 5 Division of Cancer Education, National Cancer Centre Singapore, 30 Hospital Boulevard, Singapore, 168583 Singapore; 6 Department of Internal Medicine, Singapore General Hospital, Outram Road, Singapore, 169608 Singapore; 7 Medical Social Services, Singapore General Hospital, Outram Road, Singapore, 169608 Singapore; 8 Lien Centre for Palliative Care, Duke-NUS Medical School, National University of Singapore, 8 College Road, Singapore, 169857 Singapore; 9 Duke-NUS Medical School, National University of Singapore, 8 College Road, Singapore, 169857 Singapore; 10 Palliative Care Institute Liverpool, Academic Palliative & End of Life Care Centre, University of Liverpool, 200 London Rd, Liverpool, L3 9TA UK; 11 PalC, The Palliative Care Centre for Excellence in Research and Education, PalC C/O Dover Park Hospice, 10 Jalan Tan Tock Seng, Singapore, 308436 Singapore; 12 Health Data Science, University of Liverpool, Whelan Building The Quadrangle, Brownlow Hill, Liverpool, L69 3GB UK; 13 Assisi Hospice, 832 Thomson Road, Singapore, 574627 Singapore; 14 Department of Rheumatology and Immunology, Singapore General Hospital, 16 College Road, Block 6 Level 9, Singapore, 169854 Singapore

**Keywords:** Storytelling, Narratives, Palliative Care, Physicians, Care determination, Professional identity formation, Patient centered care

## Abstract

**Background:**

Patients’ stories provide Palliative Care physicians with a glimpse into the former’s lives and their psycho-emotional, sociocultural, and contextual considerations. Yet, few physicians are trained to interpret and apply patients’ stories in their practice. Inherent variability in how stories are transmitted and interpreted raises questions over their potential effects on care. Amidst a dearth of accounts in Palliative Care, we map current use of patient stories to guide the training, assessment, and oversight of this ‘care influencing’ practice in medicine.

**Methods:**

This systematic scoping review was guided by the Systematic Evidence-Based Approach (SEBA) to ensure a reproducible and structured approach. The themes and categories identified through the Split Approach’s concurrent and independent thematic and directed content analyses provided a comprehensive sketch of the included articles. The Jigsaw Perspective combined the themes and categories identified. The last stage of SEBA compared these results with two recent reviews of storytelling to ensure consistency of the domains created that guided the discussion.

**Results:**

Ten thousand two hundred seven articles were reviewed, 963 full text articles were evaluated, and 199 articles were included. The four domains identified were study characteristics, benefits, approaches, and positive effects and concerns.

**Conclusion:**

Stories support patient-centered, personalized, and holistic clinical care. However, variability in the stories, their interpretations and use in care decisions underscore the need for further study on the structuring, teaching, assessing, and delivery of this ‘care influencing’ practice.

**Supplementary Information:**

The online version contains supplementary material available at 10.1186/s12904-023-01319-w.

## Background

Sitting at the heart of physician-patient relationships are patients’ stories that supplement physicians with rich context-sensitive, culturally-pertinent, personalized, spiritual, sociocultural, relational, and psycho-emotional information about patients and their needs, goals, preferences and interests [1–9]. Patients’ stories contain their narratives that concern the “formalized, academic version of narrative medicine” and focus on the patient’s illness, as well as their identities, values, beliefs, and goals before their illness [10–14]. Stories also encompass personal, demographical, historical and psycho-sociocultural anecdotes and accounts.

Laskow et al. [1] further suggest that a patient’s storytelling injects much needed humanistic elements to care determinations. These humanistic features counterbalance the dominant evidenced-based medicine ethos in current practice to fashion person-centered care provision [15–18]. Storytelling builds trust, nurtures relationships, and encourages shared responsibility for the patient’s care [19]. Similarly, patients’ stories that are transmitted between members of the multidisciplinary care teams foster patient-centered care [20–22] and platform multidisciplinary team reflections [19, 23–29]. A better appreciation of stories told by patients thus promises to direct personalized and timely support to patients and their families. However, with variability in the structuring, assessment, and verification of patients’ stories, the interpretation and use of ‘care influencing’ patient stories have raised concerns [22, 30–33].

As we consider “*What is known about storytelling in clinical practice?*” and *“How do physicians employ patients’ stories in clinical practice?”* in the context of Palliative Care, an exploratory search suggested a dearth of data on patients’ stories (henceforth storytelling). We then expanded our focus to review storytelling in medicine.

## Methods

To contend for the evolving nature of this sociocultural construct, we adopted Krishna’s Systematic Evidence-Based Approach (SEBA) to guide a systematic scoping review (SSR) (henceforth SSR in SEBA) of storytelling [34–39].

SEBA’s constructivist approach [40–46] and relativist lens [47–50] acknowledge storytelling as a sociocultural construct [51, 52] molded by six key elements [53]. One, individual characteristics, working styles, opportunities [54], motivations, attitudes, emotions [55], experience, skills, goals, historical, demographic [55, 56], socio-cultural [57–59], ideological, contextual and psycho-emotional features (henceforth *accounts*). Two, clinical, academic, personal, research, professional, ethical, psychosocial, emotional, cultural, organizational, societal, legal, and educational spheres of the patient and the receiving physician and healthcare professional (henceforth *contextual considerations*) [60]. Three, the learning objectives [61], goals [62, 63], timelines and professional standards [64, 65], codes of conduct, roles, responsibilities, expectations [66, 67], implicit norms [68], culture [69], artifacts, sociocultural norms and expectations and legal requirements at the practice site [70–72] (henceforth *netiquette*) [73] and its formal curriculum, approach, stages, assessment points, and mentoring support (henceforth *standards*). Four, the physician’s and the other healthcare professionals’ skills, knowledge, evolving goals, availabilities, reflective practice, motivations, levels of engagement, judgment, decisions, actions, and psycho-emotional wellbeing (henceforth *developing competencies*) [74]. Five, the nature, frequency, circumstances, and duration of interactions between patient and healthcare professionals (henceforth *interactional considerations*) [34]. Six, reflective practice and meaning making that are often informed by the healthcare professional’s reflections; clinical and practical experience; *developing competencies*; available guidance; and maturing *belief systems* (henceforth *meaning making*)*.* Story telling is also influenced by the time limitations at consults and the presence of a conducive environment for sharing.

In the face of so many considerations, this SSR in SEBA charted current accounts, insights and the impact of stories; distilled key characteristics of storytelling to formulate and summarise actionable and applicable information across different settings; and highlighted gaps in current concepts [75–82]. A SSR in SEBA’s wide reach that included grey literature was also well-placed to contend with the conflation of the terms ‘narratives’ and ‘storytelling’. However, to ensure the feasibility of this project, we focused on the study of the term ‘storytelling’ and its impact upon physicians and patients.

**Fig. 1:**
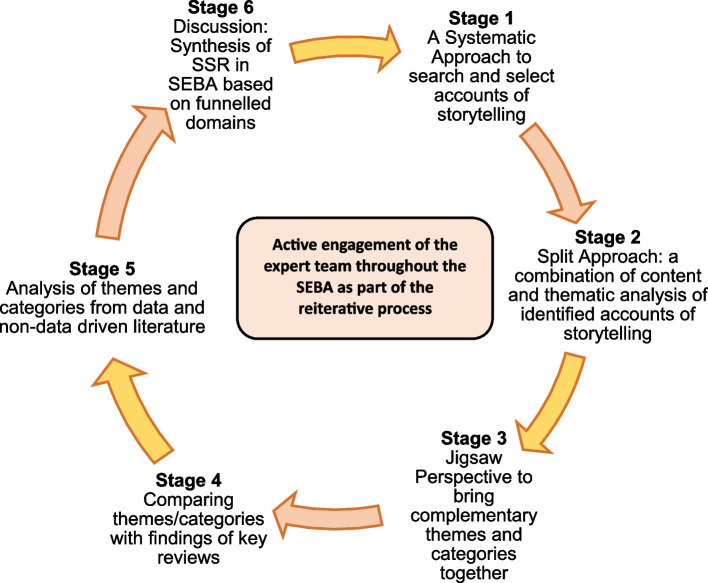
The SEBA process (*adapted from Krishna *et al*. *[79])

This SSR in SEBA involved an expert team constituting a librarian from the National University of Singapore’s (NUS) Yong Loo Lin School of Medicine (YLLSoM) and local educational experts and clinicians at YLLSoM, National Cancer Centre Singapore, Palliative Care Institute Liverpool, and Duke-NUS Medical School. Supporting SEBA methodology’s iterative process [75, 83–85], the expert team guided each stage of SEBA (Fig. 1) [38, 75, 80, 81, 85] in order to foster a balanced, reproducible and accountable review.

### Stage 1 of SEBA: systematic approach

The PCC (Population, Concept, Context) format [86] and the PRISMA-ScR checklist (see Additional file 1) were used to map the use of storytelling over the wide realm of clinical practice and guide the primary and secondary research questions [36, 37, 39, 75, 79, 81, 85]. The primary research question identified was: “*What is known about storytelling in clinical practice amongst physicians?*”. The secondary research questions identified were: *“What are the features, benefits and concerns surrounding storytelling in clinical practice?”* and *“How do physicians employ patient stories in clinical practice?”* (Table 1).

**Table 1:** PCC, inclusion and exclusion criteria applied to database search

PCC	Inclusion criteria	Exclusion criteria
Population	• Practicing physicians • Resident physicians, fellows • Patients	• Teaching faculty, master’s Programmes, higher education programmes • Allied health specialities such as pharmacy, dietetics, chiropractic, midwifery, podiatry, speech therapy, occupational and physiotherapy • Non-medical specialities such as clinical and translational Science, alternative and traditional Medicine, veterinary, dentistry • Non-medical students
Concept	• Accounts of storytelling in clinical practice	• Non-clinical settings
Context	• Clinical practice	

The populations focused on were the patients and physicians. We did not consider other audiences nor other healthcare professionals to limit the review and ensure its feasibility.

#### Searching

Independent searches of articles on storytelling in clinical practice published in PubMed, SCOPUS, ERIC, Google Scholar, Embase databases between 1^st^ January 2000 and 31^st^ December 2022 were conducted between 17^th^ January 2023 and 24^th^ April 2023. The list of titles to be reviewed was finalized during online research meetings by adopting Sandelowski and Barroso [87]’s ‘negotiated consensual validation’ [34, 75, 77, 88] that resolved discrepancies. The full search strategy may be found in Additional file 2.

### Stage 2 of SEBA: split approach

Krishna’s ‘Split Approach’ was employed to strengthen the reliability of the data analysis process [40, 87, 89–92]. This saw one team of independent researchers using Braun and Clarke [93]’s approach to thematic analysis [34, 85, 94–96] and another team of independent researchers employing Hsieh and Shannon [97]’s approach to directed content analysis to draw ‘a priori coding categories” from Laskow et al. [1]’s review [83, 84, 98–100].

#### Braun and Clarke’s thematic analysis

The first team of researchers adopted Braun and Clarke [93]’s approach to thematic analysis in independently reviewing the included articles and plotting patterns in the data. The ‘surface’ meanings of these patterns were then adapted as codes and used to create a code book. Using the code book as a guide, the team integrated each newly emerging code with past codes in an iterative step-by-step analysis process [101]. This resulted in themes that were “defined from the raw data without any predetermined classification” [96]. Discussing their independent findings, the team then determined the final list of themes through ‘negotiated consensual validation’ [87].

#### Hsieh and Shannon’s directed content analysis

Simultaneously, Hsieh and Shannon [97]’s approach to directed content analysis guided the second team of researchers in their data analysis process. Here, the researchers identified and operationalized a priori* coding categories* [97, 102–106] from Laskow et al. [1]’s review [83, 84, 98–100]. This formed the ‘coding agenda’ [106, 107] wherein the pre-established codes were used to code the included articles—reducing contradictory data and omission of negative results commonly observed in thematic analysis [38, 83, 84, 88, 98–100, 108, 109]. Data uncaptured by the priori codes were given new codes [106]. Similarly, the team reached an agreement on the final categories through ‘negotiated consensual validation’ [87].

### Stage 3 of SEBA: Jigsaw perspective

The Jigsaw Perspective entailed the merging of overlapping/complementary themes and categories identified in the Split Approach to create larger themes/categories [34, 76, 95].

### Stage 4 of SEBA: comparison

The themes/categories identified were then compared with the key findings from Laskow et al. [1] and Frioretti et al. [2]’s reviews to ensure the consistency of this data.

## Results

**Fig. 2:**
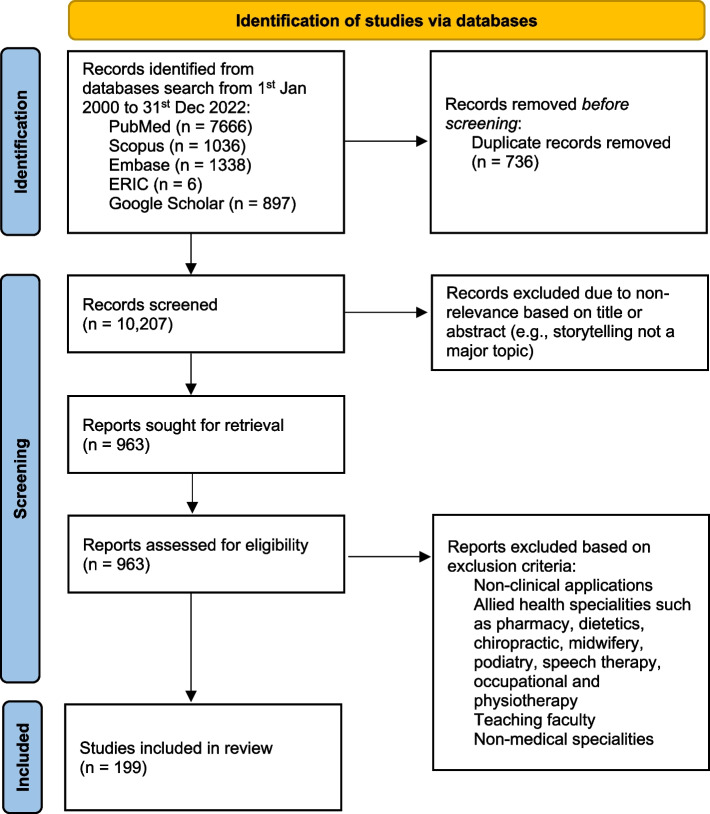
PRISMA-ScR flowchart

Of the 10,207 articles identified from the five databases, 963 full-text articles were independently reviewed, and 199 articles were included (Fig. 2). The four domains identified were study characteristics, benefits, approaches, and positive effects and concerns.

### Domain 1. Study characteristics

A total of 137 of the 199 (68.5%) publications on storytelling originated from the United States of America. The remaining contributions were derived from other regions of the world, including the United Kingdom (7.5%), Canada (5.5%) and parts of Asia (5.5%). Most accounts were situated in the post-graduate setting (53%) and took the form of author insights and reflections (24%), teaching and advice on program design (26%), use in the exploration of medical ethics (12%), shared experiences (31%), or public education (6%). Additional file 3 describes the study characteristics of the included articles in more detail.

### Domain 2. Benefits

Of the 199 included articles, 141 articles discussed the benefits of storytelling to clinicians, and 68 articles discussed benefits to patients where some articles discussed the benefits to both parties. These benefits are summarized in Table 2.

**Table 2:** Benefits of storytelling

Patient benefits	Clinician benefits
Greater psychological well-being [110, 111] through: • Reduced stress [111] • Improved destigmatization of condition [112–116] • Reduced burnout [117] • Improved validation [111]	Increased ability to attend to patients holistically [111, 114, 117–152] through: • Enhanced learning of empathy [111, 125, 127, 128, 135, 138, 145, 149, 150, 152–166] • Improved ability to understand, interpret, and engage with others [11, 19, 111, 114, 135, 155, 167–173] • Enhanced patient-centred care [111] • Enhanced building of cultural sensitivity and contextual considerations [114, 124, 130, 136, 140, 143, 170, 173–182] • Enhanced understanding of the patient’s context and concerns [30, 111, 117–119, 124, 125, 145–147, 170, 183, 184]
Enhanced physical well-being [111, 185] through: • Improved healing [186–190] • Improved symptom management [25, 28, 111, 191–194] • Higher provision of emotional and physical support [10, 111, 116, 132, 172, 195–199]	Improved clinical skills through: • Increased group interactions and attention in lessons [171, 200] • Enhanced critical thinking skills [157, 170, 182, 201, 202] • Improved framing of therapeutic plans [22, 33]
Improved individualized care through: • Enhanced shared perspectives [114, 135, 169, 171, 172, 197, 203–217] • Enhanced contextualization and personalization of care [151, 169, 194, 204, 206, 210, 212–215, 217–223]	Better physician emotional wellbeing through: • Improved ability to cope with stress and distress [12, 117, 131, 151, 152, 154, 180, 224–229] • Enhanced wellbeing [111, 114, 143, 168, 173, 201, 230] • Enhanced peer support [114, 146, 174, 195, 197, 229, 231–234] • Enhanced spiritual or existential support [235]
Championing of social justice [112, 114, 220] through: • Increased access to treatment [111, 112, 216, 236] • Improved patient advocacy [116, 183, 187, 195, 205, 220, 226, 237–250] • Improved provision of consent and information [251]	Advanced moral reasoning [114, 218, 252–256] through: • Enhanced learning of ethics [10, 112, 135, 151, 158, 252, 256–261]
	Upgraded personal development through: • Enhanced creativity [22, 149, 170, 247, 256, 262–264] • Enhanced meaning making [11, 19, 22, 24–29, 173, 180, 182, 265–268] • Enhanced self-reflection [116, 135, 139, 158, 269–272] • Enhanced professional identity formation [139, 152, 157, 202, 241, 247, 273–275]
	Enhanced development of professional common culture [139, 276] through: • Enhanced relationship building [19, 30, 111, 125, 136, 146, 148, 153, 155, 161, 192, 201, 205, 212, 271, 277–280] • Enhanced peer teaching [11, 19, 24–29, 128, 173, 182, 191] • Reduced hierarchy [12, 171, 281] • Enhanced collaborative interprofessional rapport [114, 139, 171, 174, 276]

In brief, stories helped physicians improve their patient care competencies, and enhanced moral reasoning. Stories also aided in nurturing a physician’s individual and professional development. For patients, the individual benefits of storytelling included improved psychological and physical well-being, as well as enhanced individualized care. On a societal level, the benefits to larger society extended to improved patient advocacy and personalized care.

### Domain 3. Approaches

Many current accounts structured their stories on Joseph Campbell’s hero’s journey, or the monomyth perspective (initiation, conflict, and resolution) and its subplots [265, 282–284]. These articles similarly described a hero, their initial context, their experience with a life-altering encounter that changed their lives, how they overcame challenges and were ultimately transformed [265, 282–284].

Other articles discussed the use of storytelling around Charon’s concept [19, 135, 169]. Here, these accounts echoed Charon’s posit that to be able to interpret, respond to, and be moved by patients’ stories, stories ought to have a frame (why the story was being told and its purpose), a clear timeline (both the chronological trajectory and the significance of time in the story), plot (what happened), desire (what motivated the narrator to keep telling and the audience to keep listening to the story), and tone (language and metaphor).

A number of accounts also discussed Frank [173, 285]’s approach in categorising their accounts into three forms: restitution (a plot-based story), chaos (the messy experience of illness), and quest (the lessons learned through the journey of illness). Gu [286] also suggested that storytelling revealed the dialogue between the main character (who might be the storyteller) and their roles as counsellor and listener.

Moreover, Rivkin [193] noted that stories were framed with the audience and context in mind—underlining the role, talent and experience of the storyteller, the relevance of the story to the discussion, its ability to engage the audience and the appropriateness of the content. Sandars [284, 287] further suggested that the impact of stories were enriched by its credibility that sought to balance the need for the objectivity of evidence-based medicine and the narrative subjectivity of both patients’ and clinicians’ lived experiences [131].

### Domain 4. Positive effects and concerns

The absence of formal assessments of storytelling on patients and physicians left much to be inferred. These considerations underlined the need for closer study of stories and how they were retold. Even within the multidisciplinary approach of Palliative Care settings, the risk of ill-equipped physicians misinterpreting patients’ stories and inadvertently affecting decisions on care persisted [30, 32, 33, 180, 182]. The positive and negative impacts of patients’ stories on professional, personal and interpersonal development, as well as patient-physician relationships, are summarized in Table 3.

**Table 3:** Impact of patients’ stories

Impact	
**Positive**	**Negative**
***Professional Development***	
Greater appreciation of the patient’s context, symptomology, contextual considerations, and concerns [114, 124, 130, 143, 174, 175, 180, 182]	Lack of training, support and remediation in the use of stories in clinical practice [19, 139, 153, 169]
Molding of professional identities (PIF) [139, 152, 157, 170, 202, 241, 247, 273–275, 288, 289]	Difficulty in verifying information thus risking skewing care determinations [30, 32, 33, 180, 182]
***Patient- physician Relationship***	
Enhanced patient-centered care through [22, 33, 111, 290, 291]: • Enhanced relationship building [19, 30, 111, 125, 136, 146, 148, 153, 155, 161, 192, 201, 205, 212, 271, 277–280, 292–294], • Enhanced trust [111], • Enhanced cultural sensitivity [114, 140, 170, 173, 176–179, 181, 290] • Improved communication [12, 29, 32, 33, 115, 146, 150, 154, 163, 166, 173, 176, 183, 189, 194, 228, 247, 252, 260, 267, 268, 271, 273, 279, 281, 288] • Improved framing of therapeutic plans [12, 25, 27–29, 33, 117, 150, 154, 173, 189, 192–194, 211, 227, 247, 252, 260, 268, 271, 272, 274, 276, 279, 284, 288, 293]	Risk of overwhelming emotional investment in patients thus requiring careful disentangling from emotional and psychological distress [33, 129, 157, 197]
	Lack of clear feedback and guidelines on creeping breaches in professional boundaries until professional standards are broken [29, 115, 173, 180, 188, 197, 225, 254, 272, 288]
***Personal Development***	
Enhanced self-reflection [116, 135, 139, 158, 269–272]	Poor emotional and psychological support [115, 163, 165, 173, 197, 244, 256, 268, 288]
	Lack of feedback and remediation [115, 178, 197, 256, 272]
***Interprofessional Development***	
Enhanced collaboration and interprofessional working [114, 116, 135, 139, 158, 171, 174, 269–272, 276]	
Enhanced integration of common culture [139, 276]	
Reduced hierarchy [12, 171, 281]	
Enhanced practical wisdom [12, 25, 29, 117, 154, 173, 191, 194, 211, 228, 247, 250, 273, 279, 280, 288]	

These long-term effects on the physician underscored the need for timely, personalized and longitudinal support, assessments and remediation, alongside a place for a learning portfolio to guide and mentor a physician’s professional and personal development [157, 170].

### Stage 5 of SEBA: analysis of evidence-based and non-data driven literature

With nearly half the included articles drawn from non-data-based articles (grey literature, opinion, perspectives, editorial, letters), themes from these sources were compared with themes derived from evidence-based peer-reviewed sources. This process revealed similar themes and categories in the two groups. We thus concluded that data from non-data-based articles did not introduce any untoward biases to our analysis.

## Discussion

### Stage 6 of SEBA: synthesis of discussion

In answering its primary research question, “*What is known about storytelling in clinical practice?*”, this SSR in SEBA reveals widespread albeit informal, unstructured use of storytelling in medical practice that molds the physician-patient relationship, informs clinical decisions, and shapes practice. Some appreciation as to the extent of these effects becomes apparent as we address our secondary research question, *“What are the features, benefits and concerns surrounding storytelling in clinical practice?”.* The impacts of stories are summarized in Table 3.

When considering *“How do physicians employ patient stories in clinical practice?”*, our findings do suggest that current concerns about the unguided and unverified use of stories to influence care determinations merit closer scrutiny. Indeed, by virtue of their sociocultural roots and their variable structure, content, goals, nature, chronology and even the subject matter, the threat of variability and misinterpretation of stories runs the risks of inadequate and or inappropriate patient care, mistrust, and even a break in the physician–patient relationship.

However, the benefits of storytelling cannot be understated, particularly within a clinical team. Returning to our focus in the Palliative Care setting, bringing in a multidisciplinary team with inputs from nurses, medical social workers, psychologists, physiotherapists, occupational therapists, and other members of the team provide greater depth and may help verify accounts from other sources and situations. The presence of various team members, especially nurses, psychologists, counsellors, members of the chaplaincy team and social workers, attenuates the effects posed by the absence of a formal training process for physicians on the use of stories and the lack of assessment of their ‘narrative competence’ [83, 295–298]. Here, the Palliative Care team could temper inaccuracies and paint a holistic perspective of a patient’s story. This, however, does not negate the need for training of physicians. This training should be context sensitive and appropriate to the practice settings and be focused on enhancing understanding patients and their needs over time.

Further, the Palliative Care team also serves a key role in supporting physicians and one another as they confront the effects of emotional, traumatic, and difficult stories [34, 35, 53, 81]. Indeed, whilst there was only brief mention of it in this SSR in SEBA, the potential for vicarious trauma [33, 129, 157, 197] cannot be discounted. Described as a *“transformation in the therapists’ inner experience, resulting from empathic engagement with clients’ trauma material [and] … the cumulative, transformative effect upon the trauma therapist, of working with survivors of traumatic life events…[,] a natural outcome of working with traumatised patients”*[299], Ho and colleagues reveal concerning and long term effects of vicarious trauma upon clinicians that could be assuaged with early diagnosis and support. Here, the Palliative Care team provides a platform for just support.

## Limitations

Our exclusion of healthcare professionals, such as nurses and medical social workers, to confine this review and ensure its feasibility has limited the scope and applicability of these findings. Similarly, excluding family members and care providers, particularly when taken in the Southeast Asian context where stories, care and autonomy are shared, further limits the ease of extrapolating these findings. Moreover, extrapolating accounts of storytelling from general medical settings to the Palliative Care setting also remains unproven and warrants closer scrutiny.

Including articles in or translated into English may have also restricted the search results. As most of the data were drawn from North America and Europe, they may not necessarily be transferable beyond these regions, in light of the socioculturally-sensitive nature of storytelling and stories.

## Conclusion

Given the benefits of storytelling, as well as an increasing interest and general expansion of narrative medicine within medical practice, we believe that designing a formal means of assessing and structuring stories is essential, as should be efforts to better equip physicians to listen, interpret, respond to, and be moved by patients’ stories [19, 169]. In the absence of data on the impact of cultural sensitivity [294, 300–303], contextual appreciation, vicarious trauma [33, 129, 157], and empathy [88] amongst physicians, storytelling’s sociocultural roots and individualized lens should be evaluated as a means of supporting and training for physicians. We believe this is especially relevant to Palliative Care, Rehabilitation Medicine and geriatrics. It is to this and the appreciation of storytelling in Palliative Care that we will focus our efforts as we hope to continue this discussion.

### Supplementary Information

**Additional file 1.** Preferred Reporting Items for Systematic reviews and Meta-Analyses extension for Scoping Reviews (PRISMA-ScR) Checklist.

**Additional file 2.** Search Strategies.

**Additional file 3.** Study Characteristics of Included Articles.

## Data Availability

All data generated or analysed during this review are included in this published article.

## Abbreviations

SEBA: Systematic Evidence Based Approach
SSR: Systematic Scoping Review
PCC: Population, Concept, Context
PIF: Professional Identity Formation
