# Supplementary material for: The role of patients’ stories in medicine: a systematic scoping review
Source: BMC Palliat Care. 2023 Dec 12;22:199. doi: 10.1186/s12904-023-01319-w (PMC10714554; doi:10.1186/s12904-023-01319-w)
Supplement: Supplementary file 2 — Additional file 2. Search Strategies. [file 12904_2023_1319_MOESM2_ESM.docx]

**Additional File 2. Search Strategies**

**PubMed**

| #1 | ("storytelling"[Title/Abstract] OR "story"[Title/Abstract] OR "stories"[Title/Abstract] OR "story-telling"[Title/Abstract] OR "narrative-ethics"[Title/Abstract] OR "illness experience"[Title/Abstract] OR "expressive writing"[Title/Abstract] OR "reflective writing"[Title/Abstract] OR "reflection*"[Title/Abstract] OR "creative writing"[Title/Abstract]) AND (2000/01/01:2022/12/31[Date - Publication] AND "english"[Language]) | 104,676 |
| --- | --- | --- |
| #2 | ("physicians"[MeSH Terms] OR "patients"[MeSH Terms] OR "patient*"[Title/Abstract] OR "physician"[Title/Abstract] OR "physicians"[Title/Abstract] OR "doctor"[Title/Abstract] OR "doctors"[Title/Abstract] OR "clinician"[Title/Abstract] OR "clinicians"[Title/Abstract] OR "resident"[Title/Abstract] OR "residents"[Title/Abstract] OR "medical practitioner"[Title/Abstract] OR "medical practitioners"[Title/Abstract]) AND (2000/01/01:2022/12/31[Date - Publication] AND "english"[Language]) | 5,916,783 |
| #3 | ("medicine"[MeSH Terms] OR "patient care"[MeSH Terms] OR "palliative medicine"[Title/Abstract] OR "palliative care"[Title/Abstract] OR "medicine"[Title/Abstract] OR "clinical practice"[Title/Abstract] OR "patient care"[Title/Abstract] OR "end-of-life care"[Title/Abstract]) AND ((2000/1/1:2022/12/31[pdat]) AND (english[Filter])) | 1,769,145 |
|  | #1 AND #2 AND #3 | 7666 |

**Scopus**

| TITLE-ABS-KEY ( "physicians" OR "physician" OR "doctor" OR "doctors" OR "clinician" OR "clinicians" OR "medical practitioner" OR "medical practitioners" OR "medical officer" OR "medical officers" OR "house officer" OR "house officers" OR "residents" OR "resident" OR "patient" ) W/20 TITLE-ABS-KEY ("storytelling" OR "story" OR "stories" OR "story-telling" OR "illness experience" OR "expressive writing" OR "reflective writing" OR "reflections" OR "creative writing" ) W/20 TITLE-ABS-KEY ( "medicine" OR "patient care" OR "palliative medicine" OR "palliative care" OR "end-of-life care" OR "end of life care" ) AND ( PUBYEAR > 1999 ) AND ( PUBYEAR < 2023 ) AND LANGUAGE ( english ) | 1,036 |
| --- | --- |

**Embase**

| 1 | (“physician” OR “clinician” OR “resident” OR patient)/exp OR (“physician*” OR “clinician*” OR “doctor*” OR “medical officer*” OR “attending physician*” OR “consultant*” OR “patient*”):ab,ti AND [2000-2022]/py AND [embase]/lim NOT ([embase]/lim AND [medline]/lim) AND [english]/lim | 4,363,639 |
| --- | --- | --- |
| 2. | ( “stories” OR “storytelling” OR “story-telling”):ab,ti AND [2000-2022]/py AND [embase]/lim NOT ([embase]/lim AND [medline]/lim) AND [english]/lim | 5,963 |
| 3 | (“medicine” OR “palliative therapy” OR “patient care”)/exp OR (“patient care” OR “medicine” OR “palliative” OR “end-of-life” OR “end of life” OR “clinical practice”):ab,ti AND [2000-2022]/py AND [embase]/lim NOT ([embase]/lim AND [medline]/lim) AND [english]/lim | 1,886,342 |
|  | #1 AND #2 AND #3 | 1,338 |

**ERIC**

*Filters applied: English only, 01/01/2000-31/12/2022*

| 1 | MAINSUBJECT.EXACT.EXPLODE("Physicians") OR MAINSUBJECT.EXACT("Patients") OR title(physician* OR doctor* OR medical officer* OR consultant* OR attending* OR patient*) AND abstract(physician* OR doctor* OR medical officer* OR consultant* OR attending* OR patient*) | 9,652 |
| --- | --- | --- |
| 2 | MAINSUBJECT.EXACT("Story Telling") OR MAINSUBJECT.EXACT("Narration") OR abstract("narrative medicine" OR "narratives" OR “story” OR "stories" OR "storytelling" OR "story-telling") AND title("narrative medicine" OR "narratives" OR “story” OR "stories" OR "storytelling" OR "story-telling") | 7,880 |
| 3 | (MAINSUBJECT.EXACT("Medicine") OR MAINSUBJECT.EXACT("Internal Medicine")) OR abstract("patient care" OR "medicine" OR "palliative care" OR "end of life" OR "end-of-life" OR "clinical practice") AND title("patient care" OR "medicine" OR "palliative care" OR "end of life" OR "end-of-life" OR "clinical practice") | 1,703 |
|  | #1 AND #2 AND #3 | 6 |

**Google Scholar**

*Limits: 2000-2022*

| allintitle: storytelling OR stories OR story "medicine" | 897 |
| --- | --- |
