# Supplementary material for: The role of patients’ stories in medicine: a systematic scoping review
Source: BMC Palliat Care. 2023 Dec 12;22:199. doi: 10.1186/s12904-023-01319-w (PMC10714554; doi:10.1186/s12904-023-01319-w)
Supplement: Supplementary file 3 — Additional file 3. Study Characteristics of Included Articles. [file 12904_2023_1319_MOESM3_ESM.pdf]

Additional File 3. Study Characteristics of Included Articles

Author	Journal	DOI	Year of Publication	Title	Country	Population	Definition of story telling	Document type	Undergraduate/ Graduate	Undergraduate/ Graduate	Purpose (Primary)	Purpose (Secondary)	Purpose (Tertiary)
Baikie, K.A.<br>Wilhelm, K.	Advances in Psychiatric Treatment	10.1192/apt.11.5.338	2005	Emotional and physical health benefits of expressive writing	Australia	Clinician	No	Journal Article	Undergraduate		Physical and Psychological Health	Reflections	
Fivush, R.	Journal for the Humanities in Rehabilitation	none	2017	The Power of Stories for Patients and Providers	USA	Clinician	No	Perspectives	Post-graduate		Reflections	Teaching	
Beresford, P.	Canadian Journal of Disability Studies	none	2019	Including Our Self In Struggle: Challenging the neo-liberal psycho-system's subversion of us, our ideas and action	UK	Clinician	No	Journal Article	Post-graduate		Reflections		
González-Sanguino, C.<br>Potts, L.C.<br>Milenova, M.<br>Henderson, C.	BMC Psychiatry	10.1186/s12888-019-2415-x	2019	Time to Change's social marketing campaign for a new target population: results from 2017 to 2019	UK	Non-clinician	No	Journal Article	NA		Public Education		
Harter, L. M.<br>Ellingson, L. L.<br>Yamasaki, J.<br>Hook, C.<br>Walker, T.	Health Commun	10.1080/10410236.2018.1557468	2020	Defining Moments…Telling Stories to Foster Well-being, Humanize Healthcare, and Advocate for Change.	USA	Clinician	No	Journal Article	Post-graduate		Reflections		
Kwan, C.K.	Int J Environ Res Public Health	10.3390/ijerph17093029	2020	A Qualitative Inquiry into the Human Library Approach: Facilitating Social Inclusion and Promoting Recovery	Hong Kong	Clinician	Yes	Journal Article	NA		Public Education	Reflections	
Brandeland, M. A.	JAMA	10.1001/jama.2022.2355	2022	The Labor of Story Telling	USA	Clinician	No	Perspectives	Post-graduate		Sharing experiences		
Accreditation Council for Graduate Medical Education	none	none	2022	#ACGME2022 Session Summary: Fostering Meaning and Connections through Storytelling and Written Reflection Accreditation Council for Graduate Medical Education	USA	Clinician	Yes	Blog	Post-graduate		Reflections	Sharing experiences	Teaching
UT Health San Antonio Charles E. Cheever, Jr. Center for Medical Humanities & Ethics	none	none	2021	HIV Storytelling Project: UT Health San Antonio	USA	Clinician	No	Website	Undergraduate		Teaching	Public Education	Sharing experiences
Humanities WUP	none	none	2017	Stories of Illness and Health: Western University Public Humanities	UK	Clinician and non-clinicians	No	Website	Undergraduate	Post-graduate	Public Education	Reflections	Teaching
Greene MG,<br>Hoffman S,<br>Charon R,<br>Adelman R.	Gerontologist	10.1093/geront/27.2.164	1987	Psychosocial concerns in the medical encounter: a comparison of the interactions of doctors with their old and young patients	USA	Clinician	No	Journal Article	Post-graduate		Ethics		
Charon, R.	Lit Med	10.1353/lm.2011.0271	1992	To build a case: medical histories as traditions in conflict	USA	Clinician	No	Journal Article	Post-graduate		Sharing experiences	Reflections	
Selzer, R.<br>Charon, R.	Acad Med	10.1097/00001888-199901000-00016	1999	Stories for a humanistic medicine	USA	Clinician	Conflated with Narrative Medicine	Journal Article	Post-graduate		Sharing experiences	Reflections	
Charon, R.	Med Humanit Rev	none	2002	The body and the self: the seamless experience of being	USA		Conflated with Narrative Medicine	Journal Article	Post-graduate		Reflections	Sharing experiences	
Charon, R.	N Engl J Med	10.1056/NEJMp038249	2004	Narrative and Medicine	USA	Clinician	No	Perspectives	Post-graduate		Reflections		
DasGupta S, Charon R	Acad Med	10.1097/00001888-200404000-00013	2004	Personal illness narratives: using reflective writing to teach empathy	USA	Clinician	Conflated with Narrative Medicine	Journal Article	Undergraduate		Teaching		
Pullman, D.<br>Bethune, C.<br>Duke, P.	Teach Learn Med	10.1207/s15328015tlm1703_14	2005	Narrative means to humanistic ends	Canada	Clinician	Conflated with Narrative Medicine	Journal Article	Undergraduate		Teaching	Ethics	
Wiltshire, J.	Intern Med J	10.1111/j.1445-5994.2004.00792.x	2005	Medical history from the viewpoint of the patient	Australia	Non-clinician	No	Journal Article	Post-graduate		Sharing experiences	Teaching	
Poirier, S.	Lit Med	10.1353/lm.2007.0008	2006	Medical education and the embodied physician	USA	Clinician	Yes	Journal Article	Post-graduate		Reflections		
Charon, R	Can Fam Physician	PMC1949238	2007	What to do with stories: the sciences of narrative medicine	USA	Clinician	Conflated with Narrative Medicine	Perspectives	Post-graduate		Reflections		
Madjar I, Kacen L, Ariad S, Denham J	Qual Health Res.	10.1177/1049732306298806	2007	Telling their stories, telling our stories: physicians' experiences with patients who decide to forgo or stop treatment for cancer	Israel and Australia	Clinician	No	Journal Article	Post-graduate		Sharing experiences	Ethics	
Charon, R.<br>Wyer, P.<br>Nebm Working Group	Lancet	10.1016/s0140-6736(08)60156-7	2008	Narrative evidence based medicine	USA	Clinician	No	Perspectives	Post-graduate		Reflections		
Yale Medicine Magazine	none	none	2008	A doctor's passion for medical storytelling	USA	Clinician	Yes	Editorial	Post-graduate		Sharing experiences		

Author	Journal	DOI	Year of Publication	Title	Country	Population	Definition of story telling	Document type	Undergraduate/ Graduate	Undergraduate/ Graduate	Purpose (Primary)	Purpose (Secondary)	Purpose (Tertiary)
Wood, W.	HemOnc Today	none	2009	Telling stories: our cancer patients as people	USA	Clinician	No	Perspectives	Post-graduate		Sharing experiences		
Garden, R.	Perspect Biol Med	10.1353/pbm.0.0135	2010	Telling stories about illness and disability: the limits and lessons of narrative	USA	Clinician	No	Journal Article	Post-graduate		Reflections		
Shapiro, J.<br>Elena Bezzubova, E.<br>Koons, R.	Virtual Mentor.	10.1001/virtualmentor.2011.13.7.medu1-1107	2011	Medical Students Learn to Tell Stories about Their Patients and Themselves	USA	Clinician	No	Journal Article	Undergraduate		Teaching	Reflections	
Charon, R.	N Engl J Med	10.1056/NEJMp1210787	2012	The reciprocity of recognition--what medicine exposes about self and other	USA	Clinician	No	Journal Article	Post-graduate		Reflections	Teaching	
Munn, J. C.	Am J Hosp Palliat Care	10.1177/1049909111421340	2012	Telling the story: perceptions of hospice in long-term care	USA	Clinician	No	Journal Article	Post-graduate		Public Education		
Yollin, P.	University of California San Francisco	none	2012	Physicians Emphasize Importance of Story Telling to Advance Patient Care	USA	Clinician	Yes	News	Post-graduate		Sharing experiences	Public Education	
Arntfield, S. L.<br>Slesar, K.<br>Dickson, J.<br>Charon, R.	Patient Educ Couns	10.1016/j.pec.2013.01.014	2013	Narrative medicine as a means of training medical students toward residency competencies	USA	Clinician	Conflated with Narrative Medicine	Journal Article	Undergraduate		Teaching		
Charon, R.	JAAPA	10.1097/01.JAA.0000437751.53994.94	2013	Narrative medicine: caring for the sick is a work of art	USA	Clinician	Conflated with Narrative Medicine	Perspectives	Post-graduate		Reflections	Teaching	
Sarah de Leeuw	Canadian Family Medicine	PMC3994787	2014	Telling stories about stories	Canada	Clinician	No	Perspectives	Post-graduate		Sharing experiences	Reflections	
Pethes, N.	Lit Med	10.1353/lm.2014.0013	2014	Telling cases: writing against genre in medicine and literature	USA	Clinician	No	Journal Article	Post-graduate		Reflections		
Sarvimäki, A.	Scandinavian Journal of Public Health Supplement	10.1177/1403494814568597	2015	Healthy ageing, narrative method and research ethics	Finland	Clinician	Yes	Journal Article	NA		Sharing experiences	Ethics	
Practice Builders	none	none	2017	Medical Practice Marketing: The Art and Science of Storytelling	USA	Clinician	Yes	Blog	NA		Sharing experiences		
Charon, R.	Acad Med	10.1097/ACM.0000000000001989	2017	To See the Suffering	USA	Clinician	No	Perspectives	Post-graduate		Reflections	Teaching	
Gallagher, S.	Duke Global Health Institute	none	2018	This Class Puts Stories at the Heart of Medicine and Health	USA	Clinician	Yes	News	Undergraduate		Teaching	Sharing experiences	
Lapite, A.	Wolters Kluwer	none	2020	Embracing patient stories: The benefits of narrative medicine	Netherlands	Clinician	Conflated with Narrative Medicine	Editorial	Post-graduate		Reflections	Sharing experiences	
Charon, R.	Lancet	10.1016/S0140-6736(21)02656-8	2021	Knowing, seeing, and telling in medicine	USA	Clinician	No	Perspectives	Post-graduate		Ethics	Sharing experiences	
Czerska, I.	European Research Studies Journal,	10.35808/ersj/2393	2021	Narrative Medicine as an Opportunity to Humanize Healthcare in a Post-Pandemic Reality	Poland	Clinician	Conflated with Narrative Medicine	Journal Article	Post-graduate		Reflections		
Zurzycka, P.<br>Wojtas, K.	Nursing Problems	10.5114/ppiel.2021.110454	2021	Narration in medical care. Selected aspects of narrative medicine in psychiatry	Poland	Clinician	Conflated with Narrative Medicine	Journal Article	Post-graduate		Sharing experiences	Reflections	
Llewellyn-Beardsley, J.<br>Rennick-Egglestone, S.<br>Pollock, K.<br>Ali, Y.<br>Watson, E.<br>Franklin, D.<br>Yeo, C.<br>Ng, F.<br>McGranahan, R.<br>Slade, M.<br>Edgley, A.	Qual Health Res	10.1177/10497323221118239	2022	'Maybe I Shouldn't Talk': The Role of Power in the Telling of Mental Health Recovery Stories	UK	Clinician	No	Journal Article	NA		Reflections	Ethics	
Silver, Michelle<br>Ohnigian, Sarah<br>Silk, Hugh<br>Ennis, Michael<br>Savageau, Judith	International Journal of Medical Students	10.5195/ijms.2021.1070	2022	Med Moth: A Storytelling Platform for Improving Wellness in Medical Education	USA	Clinician	No	Journal Article	Undergraduate	Post-graduate	Teaching	Sharing experiences	
Alcauskas M, Charon R	Neurology	10.1212/01.wnl.0000304945.48551.13	2008	Right brain: reading, writing, and reflecting: making a case for narrative medicine in neurology	USA	Clinician	Conflated with Narrative Medicine	Editorial	Undergraduate		Teaching		
Calman, K.	Clin Med (Lond)	10.7861/clinmedicine.1-3-227	2001	A study of storytelling, humour and learning in medicine	UK	Clinician	Yes	Journal Article	Post-graduate		Teaching	Sharing experiences	Ethics

Author	Journal	DOI	Year of Publication	Title	Country	Population	Definition of story telling	Document type	Undergraduate/ Graduate	Undergraduate/ Graduate	Purpose (Primary)	Purpose (Secondary)	Purpose (Tertiary)
Cappuccio, A.<br>Sanduzzi Zamparelli, A.<br>Verga, M.<br>Nardini, S.<br>Policreti, A.<br>Porpiglia, P. A.<br>Napolitano, S.<br>Marini, M. G.	ERJ Open Res	10.1183/23120541.00155-2017	2018	Narrative medicine educational project to improve the care of patients with chronic obstructive pulmonary disease	Italy	Clinician	Conflated with Narrative Medicine	Journal Article	Post-graduate		Teaching		
Charon R.<br>DasGupta,S.	Lit Med	10.1353/lm.2011.0329	2011	Narrative medicine, or a sense of story	USA	Clinician	Conflated with Narrative Medicine	Editorial	Post-graduate		Ethics	Sharing experiences	
Cunningham, H.<br>Taylor, D.<br>Desai, U. A.<br>Quiah, S. C.<br>Kaplan, B.<br>Fei, L.<br>Catallozzi, M.<br>Richards, B.<br>Balmer, D. F.<br>Charon, R.	Acad Med	10.1097/ACM.0000000000002102	2018	Looking Back to Move Forward: First-Year Medical Students' Meta-Reflections on Their Narrative Portfolio Writings	USA	Clinician	Conflated with Narrative Medicine	Journal Article	Undergraduate		Teaching		
Daryazadeh, S.<br>Adibi, P.<br>Yamani, N.	Med Ethics Hist Med	10.18502/jmehm.v14i21.8181	2021	The role of narrative medicine program in promoting professional ethics: perceptions of Iranian medical students	Iran	Clinician	No	Journal Article	Undergraduate		Teaching		
Fenstermacher, E.<br>Longley, R.M.<br>Amonoo, H.L.	Psychiatr Clin North Am	10.1016/j.psc.2021.03.006	2021	Finding the Story in Medicine: The Use of Narrative Techniques in Psychiatry	USA	Clinician	Conflated with Narrative Medicine	Journal Article	Post-graduate		Teaching		
Kagawa, Y.<br>Ishikawa, H.<br>Son, D.<br>Okuhara, T.<br>Okada, H.<br>Ueno, H.<br>Goto, E.<br>Tsunezumi, A.<br>Kuichi, T.	BMC Med Educ	10.1186/s12909-023-04054-1	2023	Using patient storytelling to improve medical students' empathy in Japan: a pre-post study	Japan	Clinician	Conflated with Narrative Medicine	Journal Article	Undergraduate		Teaching	Sharing experiences	
Kelsall, C.	HemOnc Today	none	2016	Physician–author passionate about listening to patients' stories, telling his own	USA	Clinician	No	News	Post-graduate		Reflections	Sharing experiences	
Kleinman, A.	Lancet	10.1016/s0140-6736(09)60087-8	2009	Caregiving: the odyssey of becoming more human	USA	Clinician	No	Perspectives	Post-graduate		Reflections		
Salter, L.<br>Newkirk, J.	Storytelling, Self, Society	10.13110/storselfsoci.15.1.0108	2019	Collective Storytelling for Health: A Three-Part Story. Storytelling, Self, Society	USA	Clinician	Yes	Journal Article	Post-graduate		Reflections	Sharing experiences	
Schwartz, B.D.<br>Horst, A.<br>Fisher, J.A.<br>Michels, N.<br>Van Winkle, L.J.	Int J Environ Res Public Health	10.3390/ijerph17072169	2020	Fostering Empathy, Implicit Bias Mitigation, and Compassionate Behavior in a Medical Humanities Course	USA	Clinician	No	Journal Article	Undergraduate		Reflections		
Sequence Health	none	none	2022	Healthcare Storytelling: Using Narratives to Transform Patient Care	USA	Clinician	Yes	Blog	Post-graduate		Sharing experiences	Teaching	
Zhao, J.<br>Xiantao, O.<br>Li, Q.<br>Liu, H.<br>Wang, F.<br>Li, Q.<br>Xu, Z.<br>Ji, S.<br>Yue, S.	BMC Med Educ	10.1186/s12909-023-04096-5	2023	Role of narrative medicine-based education in cultivating empathy in residents	China	Clinician	Conflated with Narrative Medicine	Journal Article	Post-graduate		Teaching	Sharing experiences	
Clayton J Baker; Stephanie Brown Clark	The Clinical Teacher	None	2008	Taking a history, telling a tale: a story-telling approach to teaching history-taking skills	USA	Clinician	Conflated with Narrative Medicine	Journal Article	Undergraduate		Teaching		
Carolyn Ellis	International Review of Qualitative Research	10.1525/irqr.2009.2.1.3	2009	Telling Tales on Neighbors: Ethics in Two Voices	USA	Clinician	No	Journal Article	Post-graduate		Sharing experiences	Reflections	
Charon, R.	JAMA	10.1001/jama.286.15.1897	2001	Narrative medicine: a model for empathy, reflection, profession, and trust	USA	Clinician	Conflated with Narrative Medicine	Journal Article	Post-graduate		Ethics	Sharing experiences	Reflections
Charon, R.	Oxford University Press	none	2006	Narrative medicine: Honoring the stories of illness	USA	Clinician	Conflated with Narrative Medicine	Book	Post-graduate		Sharing experiences	Ethics	Teaching

Author	Journal	DOI	Year of Publication	Title	Country	Population	Definition of story telling	Document type	Undergraduate/ Graduate	Undergraduate/ Graduate	Purpose (Primary)	Purpose (Secondary)	Purpose (Tertiary)
Charon, R.	Presse Med	10.1016/j.lpm.2012.10.015	2013	Narrative medicine in the international education of physicians	USA	Clinician	Conflated with Narrative Medicine	Journal Article	Undergraduate		Reflections	Teaching	
Charon, R.<br>Hermann, N.<br>Devlin, M. J.	Acad Med	10.1097/ACM.0000000000000827	2016	Close Reading and Creative Writing in Clinical Education: Teaching Attention, Representation, and Affiliation	USA	Clinician	No	Journal Article	Undergraduate		Teaching		
Gowda, D.<br>Curran, T.<br>Khedagi, A.<br>Mangold, M.<br>Jiwani, F.<br>Desai, U.<br>Charon, R.<br>Balmer, D.	Perspect Med Educ	10.1007/s40037-019-0497-2	2019	Implementing an interprofessional narrative medicine program in academic clinics: Feasibility and program evaluation	USA	Clinician	Conflated with Narrative Medicine	Journal Article	Undergraduate		Teaching		
Greene, M.G.<br>Adelman, R.D.<br>Charon, R.<br>Friedmann, E.	Gerontologist	10.1093/geront/29.6.808.	1989	Concordance between physicians and their older and younger patients in the primary care medical encounter	USA	Clinician	No	Journal Article	Post-graduate		Ethics		
Ingram, C.	Archives of Medicine and Health Sciences	10.4103/amhs.amhs_289_21	2021	Storytelling in medical education, clinical care, and clinician well-being	USA	Clinician	Yes	Journal Article	Post-graduate		Teaching	Sharing experiences	Reflections
Charon, R.	Narrative	10.1353/nar.2005.0017	2005	Narrative Medicine: Attention, Representation, Affiliation	USA	Clinician	No	Journal Article	Post-graduate		Reflections	Teaching	
Charon, R.<br>Fox, R.C.	JAMA	10.1001/jama.274.9.767	1995	Critiques and remedies: medical students call for change in ethics teaching	USA	Clinician	No	Commentary	Undergraduate		Ethics	Teaching	
DasGupta, S.<br>Meyer, D.<br>Calero-Breckheimer, A.<br>Costley, A.W.<br>Guillen, S.	Teach Learn Med	10.1207/s15328015tlm1801_4	2006	Teaching cultural competency through narrative medicine: intersections of classroom and community	USA	Clinician	Conflated with Narrative Medicine	Journal Article	Post-graduate		Teaching	Sharing experiences	
Kamaka, M.L.	Hawaii Med J	none	2010	Designing a cultural competency curriculum: asking the stakeholders	USA	Clinician	No	Journal Article	Undergraduate	Post-graduate	Teaching		
Kumagai, A.K.<br>Lypson, M.L.	Acad Med	10.1097/ACM.0b013e3181a42398	2009	Beyond cultural competence: critical consciousness, social justice, and multicultural education	USA	Clinician	No	Journal Article	Undergraduate		Teaching	Reflections	Sharing experiences
Kyratzis, A.	Human Development	10.1159/000085517	2005	Language and Culture Socialization through Personal Story-Telling Practice	USA	Non-clinician	Yes	Commentary	NA		Teaching	Sharing experiences	
Saffran, L.	Lancet	10.1016/S0140-6736(21)00841-2	2021	Public health storytelling practice	USA	Clinician	Yes	Perspectives	Post-graduate		Public Education	Ethics	Reflections
Shapiro, J.<br>Lie, D.<br>Gutierrez, D.<br>Zhuang, G.	BMC Med Educ	10.1186/1472-6920-6-31	2006	"That never would have occurred to me": a qualitative study of medical students' views of a cultural competence curriculum	USA	Clinician	No	Journal Article	Undergraduate		Teaching	Reflections	
Zarei, A.<br>Mojtahedzadeh, R.<br>Mohammadi, A.<br>Sandars, J.<br>Hossein Emami, S.A.	Ann Med Surg (Lond)	10.1016/j.amsu.2021.102528	2021	Applying digital storytelling in the medical oncology curriculum: Effects on students' achievement and critical thinking	Iran	Clinician	Yes	Journal Article	Undergraduate		Teaching	Reflections	
Hammer, R.R.<br>Rian, J.D.<br>Gregory, J.K.<br>Bostwick, J.M.<br>Barrett Birk, C.<br>Chalfant, L.<br>Scanlon, P.D.<br>Hall-Flavin, D.K	Med Humanit	10.1136/jmh.2010.006429	2011	Telling the patient's story: using theatre training to improve case presentation skills	USA	Clinician	Yes	Journal Article	Undergraduate		Teaching	Reflections	
Fins, J. J.	Camb Q Healthc Ethics	10.1017/S0963180116000323	2016	Giving Voice to Consciousness	USA	Clinician	No	Journal Article	Post-graduate		Reflections		
Charon, R.	Acad Med	10.1097/00001888-200001000-00008	2000	Literature and Medicine: Origins and Destinies	USA	Clinician	No	Perspectives	Post-graduate		Reflections		
Frattaroli, J.	Psychol Bull	10.1037/0033-2909.132.6.823	2006	Experimental disclosure and its moderators: a meta-analysis	USA	Non-clinician	No	Journal Article	NA		Physical and Psychological Health	Reflections	
Charon, R.	Teach Learn Med	10.1080/10401330902901748	2009	Commentary on "Creative Expressive Encounters in Health Ethics Education: Teaching Ethics as Relational Engagement"	USA	Clinician	No	Commentary	Undergraduate		Ethics	Teaching	

Author	Journal	DOI	Year of Publication	Title	Country	Population	Definition of story telling	Document type	Undergraduate/ Graduate	Undergraduate/ Graduate	Purpose (Primary)	Purpose (Secondary)	Purpose (Tertiary)
Greene, M.G.<br>Adelman, R.<br>Charon, R.<br>Hoffman, S.	Lang Commun	10.1016/0271-5309(86)90010-8 Cite	1986	Ageism in the medical encounter: an exploratory study of the doctor-elderly patient relationship	USA	Clinician	No	Journal Article	Post-graduate		Ethics		
Inui, T.S.<br>Frankel, R.M.	Acad Med	10.1097/01.ACM.0000222272.90705.ef	2006	Hello, stranger: building a healing narrative that includes everyone	USA	Clinician	No	Commentary	Post-graduate		Sharing experiences		
Robinson, A.	none	none	2014	U.S. Department of Veterans Affairs	USA	Clinician	Conflated with Narrative Medicine	Website	Post-graduate	Undergraduate	Reflections	Sharing experiences	Teaching
Simpson-Gervin, V.	Brill	10.1163/9789004396067_003	2019	Narrative Medicine and Storytelling: An Alternative Method for Healing	USA	Clinician	Conflated with Narrative Medicine	Book	Post-graduate		Reflections	Sharing experiences	
Charon, R<br>DasGupta, Sayantani<br>Hermann, Nellie<br>Irvine, Craig<br>Marcus, Eric R.<br>Rivera Colsn, Edgar<br>Spencer, Danielle<br>Spiegel, Maura	Book: online end, Oxford Academic	10.1093/med/9780199360192.003.0014	2016	The Principles and Practice of Narrative Medicine: Chapter 13	USA	Clinician	No	Book	Post-graduate		Teaching		
Holiday, J.M.	none	none	2013	Why Do Stories Matter? : Stories are Medicine	USA	Clinician	No	Blog	Post-graduate		Public Education	Sharing experiences	Reflections
Roscoe, L. A.	Narrat Inq Bioeth	10.1353/nib.2012.0006	2012	Healing the physician's story: a case study in narrative medicine and end-of-life care	USA	Clinician	Yes	Journal Article	Post-graduate		Ethics	Reflections	
Tait, G.R.<br>Hodges, B.D.	Adv Health Sci Educ Theory Pract	10.1007/s10459-012-9411-y	2013	Residents learning from a narrative experience with dying patients: a qualitative study	Canada	Clinician	Conflated with Narrative Medicine	Journal Article	Post-graduate		Teaching	Sharing experiences	Reflections
D'Or Institute for Research and Education	none	none	2021	Storytelling reduces pain and stress, and increases oxytocin in hospitalized children	Brazil	Non-clinician	Yes	News	NA		Physical and Psychological Health	Sharing experiences	
Chen, P.W.	The New York Times	none	2011	When Patients Share Their Stories, Health May Improve	USA	Clinician	Yes	Perspectives	Post-graduate		Sharing experiences	Physical and Psychological Health	
Shankar, P.R.	Janaki Medical College Journal of Medical Science	none	2020	Using 'stories' in Nepalese medical schools.	Nepal	Clinician	Conflated with Narrative Medicine	Editorial	Undergraduate	Post-graduate	Sharing experiences	Reflections	teaching
Bell, B.	Acquity Insights	none	2022	How Storytelling Benefits the Mental Health of Medical Students	USA	Clinician	Yes	Blog	Undergraduate		Sharing experiences	Reflections	
Miller, E.<br>Balmer, D.<br>Hermann, N.<br>Graham, G.<br>Charon, R.	Acad Med	10.1097/ACM.0000000000000098	2014	Sounding narrative medicine: studying students' professional identity development at Columbia University College of Physicians and Surgeons	USA	Clinician	No	Journal Article	Undergraduate		Teaching	Reflections	
Jáuregui-Lobera, I.<br>Martínez-Gamarra, M.<br>Montes-Martínez, M.<br>Martínez-Quiñones, J.	Journal of Negative and No Positive Results	10.19230/jonnpr.3488	2020	Storytelling as instrument of communication in health contexts	Spain	clinician	Yes	Journal Article	Undergraduate	Post-graduate	Sharing experiences	Reflections	Physical and Psychological Health
Nash, W.<br>Erondu, M.<br>Childress, A.	J Med Humanit	10.1007/s10912-022-09779-6	2023	Expanding Narrative Medicine through the Collaborative Construction and Compelling Performance of Stories	USA	Clinician	Conflated with Narrative Medicine	Journal Article	Undergraduate	Post-graduate	Teaching		
Anyaegbunam, J.A. ,<br>Sotsky, J.<br>Salib,C.<br>Kissler, M.J.<br>Jiao, J.M.<br>Charon, R	JAMA	10.1001/jama.2013.283712	2013	A piece of my mind. Five voices, one story	USA	Clinician	Conflated with Narrative Medicine	Opinion	Undergraduate		Teaching	Ethics	Reflections
BMJ	BMJ	10.1136/bmj.321.7272.0	2000	Telling stories and listening to them	UK	Clinician	No	Editorial	Post-graduate		Ethics		
Cappuccio, A.<br>Napolitano, S.<br>Menzella, F.<br>Pellegrini, G.<br>Policreti, A.<br>Pelaia, G.<br>Porpiglia, P. A.<br>Marini, M. G.<br>Sound, Group	Multidiscip Respir Med	10.1186/s40248-019-0190-7	2019	Use of narrative medicine to identify key factors for effective doctor-patient relationships in severe asthma	Italy	Clinician	Conflated with Narrative Medicine	Journal Article	Post-graduate		Teaching		
Charon, R.	Partial Answers: Journal of Literature and the History of Ideas	10.1353/pan.0.0116	2006	Narrative Lights on Clinical Acts: What We, Like Maisie, Know	USA	Clinician	Conflated with Narrative Medicine	Journal Article	Post-graduate		Sharing experiences	Reflections	
Charon, R.	Acad Med	10.1097/ACM.0b013e3182628d6f	2012	Commentary: our heads touch: telling and listening to stories of self	USA	Clinician	No	Editorial	Undergraduate		Reflections	Teaching	

Author	Journal	DOI	Year of Publication	Title	Country	Population	Definition of story telling	Document type	Undergraduate/ Graduate	Undergraduate/ Graduate	Purpose (Primary)	Purpose (Secondary)	Purpose (Tertiary)
Clements, S. Coniglio, F. Mackenzie, L.	Community Ment Health J	10.1007/s10597-019-00465-x	2020	"I'm Not Telling an Illness Story. I'm Telling a Story of Opportunity": Making Sense of Voice Hearing Experiences	Australia	Clinician	No	Journal Article	NA		Sharing experiences		
Cleveland, E.F.	Acad Med	10.1097/01.ACM.0000225244.38638.0d	2006	Telling the stories of the Health Professions Partnership Initiative	USA	Clinician	No	Journal Article	Post-graduate		Teaching		
DasGupta S	Pediatrics	https://doi.org/10.1542/peds.2006-2619	2007	Between stillness and story: lessons of children's illness narratives	USA	Clinician	No	Journal Article	Post-graduate		Teaching	Reflections	
Davenport, N.H.M.	Social Science & Medicine	https://doi.org/10.1016/j.socscimed.2011.01.036	2011	Medical residents' use of narrative templates in storytelling and diagnosis	USA	Clinician	Yes	Journal Article	Post-graduate		Teaching		
Divinsky M.	Canadian Family Physician		2007	Stories for life: introduction to narrative medicine	Canada	Clinician	No	Commentary	Post-graduate		Sharing experiences		
Heriserman, A., Spiegel, M.	Lit Med	10.1353/lm.2007.0018 Cite	2006	Narrative permeability: crossing the dissociative barrier in and out of films	USA	Non-clinician	No	Journal Article	NA		Sharing experiences	Reflections	
Irvine, C.	Lit Med	https://doi.org/10.1353/lm.2005.0027	2005	The other side of silence: Levinas, medicine, and literature	USA	Clinician	No	Journal Article	Post-graduate		Ethics		
Marcus, E.R.	Int J Psychoanal	http://dx.doi.org/10.1516/PFUF-D9NF-NKHU-9QTH	2003	Medical student dreams about medical school: the unconscious developmental process of becoming a physician	USA	Clinician	No	Journal Article	Undergraduate	Post-graduate	Sharing experiences		
Slade M., Sweeney A.	World Psychiatry	https://doi.org/10.1002/wps.20783	2020	Rethinking the concept of insight	UK	Clinician	No	Journal Article	Post-graduate		Sharing experiences		
Stanley, P.H	Lit Med	https://doi.org/10.1353/lm.2005.0016	2004	The patient's voice: a cry in solitude or a call for community	USA	Non-clinician	No	Journal Article	NA		Sharing experiences		
Arras J.D.	J Med Philos	https://doi.org/10.1093/jmp/16.1.29	1991	Getting down to cases: the revival of casuistry in bioethics	USA	Non-clinician	No	Journal Article	NA		Ethics		
Chin N.P.	Families, Systems, & Health	https://psycnet.apa.org/doi/10.1037/1091-7527.23.3.307	2005	Telling an Ongoing Story With an Ethnographic Voice: Reflections on "Family, Ethnicity, and Chronic Disease: An Ongoing Story With a New Twist"	USA	Non-clinician	No	Commentary	NA		Sharing experiences		
Fisher, P., Lees, J.	Health (London)	https://doi.org/10.1177/1363459315600774	2016	Narrative approaches in mental health: Preserving the emancipatory tradition	UK	Clinician	Conflated with Narrative Medicine	Journal Article	Post-graduate		Teaching		
Hyden, L.C., Oruly, L.	Journal of Aging Studies	https://doi.org/10.1016/j.jaging.2008.01.001	2009	Narrative and identity in Alzheimer's disease: A case study	Sweden	Non-clinician	Yes	Journal Article	NA		Sharing experiences		
Jackson D.	Clin Case Rep	https://doi.org/10.1002%2Fccr3.100	2014	Telling our stories of practice through the genre of the case report	USA	Clinician	Yes	Editorial	Post-graduate		Teaching	Reflections	
Tappan, M.B.	Human Development	https://doi.org/10.1159/000288209	2010	Telling Moral Stories: From Agency to Authorship Commentary on Pasupathi and Wainryb	USA	Non-clinician	No	Commentary	NA		Reflections		
Tucker, R.P., Haydel, R., Zielinski, M., Niederkrotenthaler, T.	Journal of American College Health	https://doi.org/10.1080/07448481.2020.1767110	2020	Storytelling of suicide attempt recovery and its relationship with mental health treatment-seeking attitudes and behaviors: An experimental study	USA	Non-clinician	Yes	Journal Article	Undergraduate		Public Education	Sharing experiences	
De Vito, B., Castagno, E., Garrone, E., Tardivo, I., Conti, A., Luciani, M., Urbino, A.F., Garrino, L., Dimonte, V.	Reflective Practice	https://doi.org/10.1080/14623943.2021.2013190	2021	Narrating care during the COVID-19 pandemic in a paediatric emergency department	Italy	Clinician	No	Journal Article	Post-graduate		Sharing experiences	Reflections	
Erogul M, Chung A, Schiller J	Curr Res Emerg Med	none	2022	Formalized Storytelling in Emergency Medicine	USA	Clinician	Yes	Journal Article	Post-graduate		Teaching		
Ofri D.	Acad Med	10.1097/ACM.0000000000000672	2015	The passion and the peril: storytelling in medicine	USA	Clinician	No	Journal Article	Post-graduate		Sharing experiences		
Spector-Mersel, G., Knaifel, E.	Journal of Mental Health	https://doi.org/10.1080/09638237.2017.1340607	2017	Narrative research on mental health recovery: two sister paradigms	Israel	Clinician	Conflated with Narrative Medicine	Journal Article	NA		Sharing experiences		
Stephens, S.	Los Angeles Times	none	2020	How doctors use stories to help them cope with the coronavirus crisis	USA	Clinician	Yes	Online Article	Post-graduate	Post-graduate	Teaching		
Colwell, J.	ACP Internist	none	2020	Narrative medicine seeks the story behind the illness	USA	Clinician	No	Online Article	Post-graduate		Sharing experiences	Reflections	
Sapega, S.	Camb Q Healthc Ethics	none	2021	Listening Lab: Harnessing the Power of Storytelling	USA	Non-clinician	Yes	Online Article	NA		Sharing experiences	Public Education	Ethics
Bajaj, N., Phelan, J., McConnell, E.E., Reed, S.M.	Annals of Medicine	https://doi.org/10.1080/07853890.2023.2185674	2023	A narrative medicine intervention in pediatric residents led to sustained improvements in resident well-being	USA	Clinician	No	Journal Article	Post-graduate		Sharing experiences		

Author	Journal	DOI	Year of Publication	Title	Country	Population	Definition of story telling	Document type	Undergraduate/ Graduate	Undergraduate/ Graduate	Purpose (Primary)	Purpose (Secondary)	Purpose (Tertiary)
Alderson, T. St J., Bateman, H.	Medical Teacher	https://doi.org/10.1080/01421590216083	2009	Doctors telling stories: the place of anecdote in GP registrar training	UK	Clinician	No	Short communications	Post-graduate		Teaching		
Moran G.S., Russinova, Z., Gidugu, V., Yim, J.Y., Sprague, C.	Qual Health Res	https://doi.org/10.1177/1049732311420578	2012	Benefits and mechanisms of recovery among peer providers with psychiatric illnesses	USA	Non-clinician	No	Journal Article	NA		Sharing experiences	Physical and Psychological Health	
Oakley, S., Grealish, L., Coyne, E.	Eur J Oncol Nurs	https://doi.org/10.1016/j.ejon.2020.101793	2020	Telling their story: A qualitative descriptive study of the lived experience of expatriate palliative care nurses in the United Arab Emirates	UAE	Yes	No	Journal Article	NA		Sharing experiences		
Truong, C., Gallo, J., Roter, D., Joo., J.	Patient Educ Couns	https://doi.org/10.1016/j.pec.2019.02.006	2019	The role of self-disclosure by peer mentors: Using personal narratives in depression care	USA	Non-clinician	No	Journal Article	NA		Sharing experiences		
Wimberly, E.P.	Journal of Health Care for the Poor and Undeserved	https://doi.org/10.1353/hpu.2011.0103	2011	Story telling and managing trauma: health and spirituality at work	USA	Clinician	No	Journal Article	Post-graduate		Reflections		
Williams A., Fossey, E., Farhall, J., Foley, F., Thomas, N.	JMIR Mental Health	http://dx.doi.org/10.2196/mental.9934	2018	Recovery After Psychosis: Qualitative Study of Service User Experiences of Lived Experience Videos on a Recovery-Oriented Website	Australia	Non-clinician	No	Journal Article	NA		Sharing experiences	Public Education	
Abdel-Aziz, S., Galal, Y.S., Al Hanafy, S.H., Ghamrawy, M., Shaheen, D.S.M.	Public Health Education and Training	https://doi.org/10.3889/oamjms.2022.7382	2022	Digital Storytelling: A Video-based Approach for Engaging University Students in Health Education	Egypt	Non-clinician	As digital storytelling	Journal Article	Undergraduate		Public Education		
Moreau K.A., Eady, K., Sikora, L. and HorsleyT.	BMC Medical Education	https://doi.org/10.1186/s12909-018-1320-1	2018	The physician-elderly patient-companion triad in the medical encounter: the development of a conceptual framework and research agenda	Canada	Non-clinician		Journal Article	Post-graduate	Undergraduate	Teaching	Sharing experiences	Reflections
Bruno, R., Andrews, A., Garvey, B., Huntoon, K., Mazumder, R., Olson, J., Sanders, D., Weinbaum, I., Gorman, P.	PLOS One	https://doi.org/10.1371/journal.pone.0051827	2012	Videotaped patient stories: impact on medical students' attitudes regarding healthcare for the uninsured and underinsured	USA	Clinician	No	Journal Article	Undergraduate		Sharing experiences		
Charon R.	Lit Med	https://doi.org/10.1353/lm.2011.0295	1986	To render the lives of patients	USA	Non-clinician	No	Journal article	Undergraduate		Sharing experiences	Teaching	
Cueva, M., Kuhnley, L.J., Revels, K.C., Dignan, M., Lanier, A.P.	International Journal of Circumpolar Health	https://doi.org/10.3402/ijch.v72i0.20717	2013	Bridging storytelling traditions with digital technology	USA	Non-clinician	As digital storytelling	Journal article	NA	Post-graduate	Public Education		
Kempson D., Murdock, V.	Death Studies	https://doi.org/10.1080/07481181003765402	2009	Memory keepers: a narrative study on siblings never known	USA	Non-clinician	No	Journal Article	NA		Reflections		
Kerr, D.J.R., Deane, F.P., Crowe, T.P.	Qualitative Health Research	10.1177/1049732319886285	2020	A Complexity Perspective on Narrative Identity Reconstruction in Mental Health Recovery	Australia	Non-clinician	No	Journal Article	NA		Reflections	Sharing experiences	
Leeder, S.	Med J Aust	10.5694/mja15.c0302	2015	Telling the story of mental health	USA	Clinician	No	Journal Article	Post-graduate		Public Education		
Llewellyn- Beardsley, J., Rennick-Egglestone, S., Callard, F., Crawford, P., Farkas, M., Hui, A., Manley, D., McGranahan, R., Pollock, K., Ramsay, A., Saeler, K.T., Wright, N., Slade, M.	PLOS One	https://doi.org/10.1371/journal.pone.0214678	2019	Characteristics of mental health recovery narratives: Systematic review and narrative synthesis	UK	Non-clinician	Yes	Journal Article	NA		Sharing experiences	Reflections	
Mancini, M.A.	Qualitative Health Research	https://doi.org/10.1177/1049732318821689	2019	Strategic Storytelling: An Exploration of the Professional Practices of Mental Health Peer Providers	USA	Non-clinician	Yes	Journal Article	NA		Sharing experiences	Public Education	
Moreau, K. A.<br>Eady, K.<br>Sikora, L.<br>Horsley, T.	BMC Med Educ	10.1186/s12909-018-1320-1	2018	Digital storytelling in health professions education: a systematic review	Canada	Clinician	No	Journal Article	Undergraduate		Teaching		
Rennick-Egglestone, S., Ramsay, A., McGranahan, R., Llewellyn-Beardsley, J., Hui, A., Pollock, K., Repper, J., Yeo, C., Ng, F., Roe, J., Gillard, S., Thornicroft, G., Booth, S., Slade, M.	PLOS One	https://doi.org/10.1371/journal.pone.0226201	2019	The impact of mental health recovery narratives on recipients experiencing mental health problems: Qualitative analysis and change model	UK	Non-clinician	Conflated with Narrative Medicine	Journal Article	NA		Sharing experiences	Reflections	
Simons, S.	Emergency Medicine News	none	2018	ER Goddess: Storytelling, Advocacy, and Being Authentic	USA	Clinician	No	Online Article	Post-graduate		Sharing experiences		

Author	Journal	DOI	Year of Publication	Title	Country	Population	Definition of story telling	Document type	Undergraduate/ Graduate	Undergraduate/ Graduate	Purpose (Primary)	Purpose (Secondary)	Purpose (Tertiary)
Sisson, G.<br>Kimport, K.	Contraception	https://doi.org/10.1016/j.contraception.2013.12.015	2014	Telling stories about abortion: abortion-related plots in American film and television, 1916-2013	USA	Clinician	No	Journal Article	NA		Public Education	Sharing experiences	
Sabaretnam, M., Bothra, S., Warsi, D.	Annals of Medicine and Surgery	https://doi.org/10.1016/j.amsu.2019.03.006	2019	The technique of story-telling in thyroid diseases including surgery; useful or not	India	Non-clinician	Yes	Journal Article	NA		Teaching		
Avrahami, E.	Lit Med	10.1353/lm.2011.0327	2011	Positive wrongdoings: reading doctors' narratives on ordinary ethics	USA	Clinician	No	Journal Article	Post-graduate		Ethics		
Flagler E	Ann R Coll Physicians Surg Can	none	1997	Narrative ethics: a means to enrich medical education	Canada	Clinician	No	Journal Article	Undergraduate	Post-graduate	Ethics	Reflections	Teaching
Hoffmaster, B.	Hastings Center Report	10.1002/hast.303	2014	From Applied Ethics to Narrative Ethics: The Rationality and Morality of Telling Stories in Bioethics	Canada	Non-clinician	No	Report	NA		Sharing experiences		
Ross, J.W.	Hastings Center Report	none	1994	Literature, bioethics, and the priestly physician	USA	Clinician	No	Report	Undergraduate	Post-graduate	Ethics	Sharing experiences	
Zaner, R. M.	J Med Philos	10.1080/03605310600806117	2006	On evoking clinical meaning	USA	Clinician	No	Perspectives	Post-graduate		Reflections	Ethics	
Brody, H.	Lit Med	10.1353/lm.2011.0169	1994	"My story is broken; can you help me fix it?" Medical ethics and the joint construction of narrative	USA	Clinician	Yes	Journal Article	Post-graduate		Sharing experiences	Ethics	
Charon, R.	Ann Intern Med	10.7326/0003-4819-134-1-200101020-00024	2001	Narrative medicine: form, function, and ethics	USA	Clinician	Conflated with Narrative Medicine	Journal Article	Post-graduate		Sharing experiences	Reflections	
Charon, R.	American Journal of Bioethics	10.1162/152651601750079186	2001	What Narrative Competence is For	USA	Clinician	Yes	Journal Article	Post-graduate		Ethics		
Charon, R.<br>Brody, H.<br>Clark, MW.<br>Davis, D.<br>Martinez, R.<br>Nelson, RM.	J Med Philos	10.1093/jmp/21.3.243	1996	Literature and ethical medicine: five cases from common practice	USA	Clinician	Yes	Journal Article	Post-graduate		Ethics		
Murphy, J.W.	Perm J	https://doi.org/10.7812/TPP/19.029	2020	Ethical Considerations Related to Narrative Medicine	USA	Clinician	No	Journal Article	Post-graduate		Ethics		
Hewson, J.<br>Danbrook, C.<br>Sieppert, J.	CIER	none	2015	Engaging Post-Secondary Students And Older Adults In An Intergenerational Digital Storytelling Course Contemporary Issues in Education Research (CIER)	Canada	Non-clinician	Yes	Journal Article	Undergraduate		Ethics		
Stacy, R.<br>Spencer, J.	Med Educ	10.1046/j.1365-2923.1999.00454.x	1999	Patients as teachers: a qualitative study of patients' views on their role in a community-based undergraduate project	UK	Clinician	No	Perspectives	Undergraduate		Sharing experiences		
Walsh, CA.<br>Shier, ML.<br>Sitter, KC.<br>Sieppert, JD.	Canadian Journal for the Scholarship of Teaching and Learning.	10.5206/cjsotl-rcacea.2010.2.3	2010	Applied Methods of Teaching about Oppression and Diversity to Graduate Social Work Students: A Case Example of Digital Stories	Canada	Non-clinician	Yes	Journal Article	Undergraduate		Teaching	Sharing Experiences	
Bohanon, M.	INSIGHT Into Diversity	none	2019	The Evolving Field of Narrative Medicine Reaches the 'Core of the Human Condition'	USA	Clinician	No	Online Article	Post-graduate	Undergraduate	Sharing experiences		
N.A.	N.A.	None		Storytelling in medical education	USA	Clinician	Yes	Online Article	Post-graduate		Sharing experiences	Teaching	Public Education
Krisberg, K.	AAMC	none	2017	Narrative Medicine: Every Patient Has a Story	USA	Clinician	No	Online Article	Undergraduate		Reflections		
Mehrlich, K.D., Wasmuth, S.	American Occupational Therapy Association	none	2019	Tell My Story: Narrative Medicine as a Unique Approach to Forensic Mental Health Intervention	USA	Non-clinician	No	Online Article	NA		Public Education		
Samuel, S.	Vox	none	2020	This doctor is taking aim at our broken medical system, one story at a time	USA	Clinician	Conflated with Narrative Medicine	Online Article	Post-graduate		Sharing experiences		
Thibodeau, P.H., Boroditsky, L.	PLOS One	10.1371/journal.pone.0016782	2011	Metaphors We Think With: The Role of Metaphor in Reasoning	USA	Non-clinician	No	Journal Article	NA		Reflections		
Versalovic, E.	Baylor College of Medicine Blog Network	none	2016	Baylor Narrative Medicine's 'Off Script' storytelling hour 2016	USA	Clinician	No	Online Article	Post-graduate		Reflections		
Wong C.	Very Well Health	none	2020	How Narrative Medicine Might Benefit You	USA	Non-clinician	Yes	Online Article	NA		Sharing experiences		
Zaharias G.	Canadian Family Medicine	None	2018	What is narrative-based medicine? Narrative-based medicine	Australia	Clinician	Yes	Journal Article	Post-graduate		Teaching		

Author	Journal	DOI	Year of Publication	Title	Country	Population	Definition of story telling	Document type	Undergraduate/ Graduate	Undergraduate/ Graduate	Purpose (Primary)	Purpose (Secondary)	Purpose (Tertiary)
Zitter J.	Harvard Business Review	None	2018	How Storytelling Can Help Young Doctors Become More Resilient	USA	Clinician	No	Online Article	Post-graduate		Teaching	Reflections	
Charon, R.	The Henry James Review	10.1353/hjr.0.0015	2008	A Momentary Watcher, or the Imperiled Reader of "A Round of Visits"	USA	Clinician	No	Perspectives	NA		Reflections		
Charon, R.<br>Hermann, N.	Acad Med	10.1097/ACM.0b013e31823a59c7	2012	Commentary: a sense of story, or why teach reflective writing?	USA	Clinician	Conflated with Narrative Medicine	Editorial	Undergraduate		Reflections	Teaching	
Charon R.	Hastings Center Report	10.1002/hast.264	2014	Narrative Reciprocity	USA	Clinician	No	Report	Post-graduate		Sharing experiences	Reflections	
Lim, J.Y., Ong, S.Y.K., Ng, C.Y.G., Chan, K.L.E., Wu, S.Y.E.A., So, W.Z., Tey, G.J.C., Lam, Y.X., Gao, N.L.X., Lim, Y.X., Tay, R.Y.K, Leong, I.T.Y., Nur Diana, A.R., Chiam, M., Lim, C., Phua, G.L.G., Murugam, V., Ong, E.K. and Krishna, L.R.K	BMC Medical Education	https://doi.org/10.1186/s12909-022-03924-4	2023	A systematic scoping review of reflective writing in medical education	Singapore	Non-clinician	No	Journal Article	Post-graduate	Post-graduate	Reflections	Teaching	
Melany, C., Kuhnley, R., Lanier, A., Dignan, M., Revels, L., Schoenberg, N.E., Cueva K.	International Journal of Indigenous Health	10.18357/ijih111201616013	2016	Promoting Culturally Respectful Cancer Education Through Digital Storytelling	USA	Non-clinician	About digital storytelling	Journal Article	Post-graduate		Sharing experiences	Reflections	
Fins, J. J.	Camb Q Healthc Ethics	10.1017/S0963180120000389	2020	Two Patients: Professional Formation before "Narrative Medicine"	USA	Clinician	Conflated with Narrative Medicine	Journal Article	Undergraduate		Reflections		
Sibbald, B.	CMAJ	10.1503/cmaj.160753	2016	A clearing for narrative practice	Canada	Clinician	No	Perspectives	Post-graduate		Sharing experiences	Reflections	
Charon, R	Acad Med	10.1097/ACM.0b013e3181dc1ead	2010	Commentary: calculating the contributions of humanities to medical practice-motives, methods, and metrics	USA	Clinician	No	Perspectives	Undergraduate		Teaching	Reflections	
Boeykens, D., Boeckxstaens, P., Van de Velde, D., De Vriendt, P.	International Journal of Intergrated Care	doi.org/10.5334/ijic.ICIC22120	2022	"It is about telling my story in a trustful relationship" Learning about goal-oriented care through the experiences of patients with chronic conditions or multimorbidity	Belgium	Non-clinician	No	Conference Abstract	NA		Teaching		
Charon, R	Ann Intern Med	None	2000	Medicine, the novel, and the passage of time	USA	Clinician	No	Journal Article	Post-graduate		Reflections		
Charon, R.	Clin Geriatr Med.	10.1016/s0749-0690(05)70006-9.	2000	The seasons of the patient-physician relationship	USA	Clinician	No	Journal Article	Post-graduate		Reflections		
Hurwitz, B.<br>Charon, R.	Lancet	10.1016/S0140-6736(13)61129-0	2013	A narrative future for health care	USA	Clinician	No	Perspectives	Post-graduate		Reflections	Public Teaching	
Warmington S., McColl, G.	Adv in Health Sci Educ	10.1007/s10459-016-9689-2	2017	Medical student stories of participation in patient care-related activities: the construction of relational identity	Australia	Non-clinician	No	Journal Article	Undergraduate		Teaching	Reflections	Sharing experiences
Willis S.	British Geriatrics Society	none	2018	Life, Death and the Stories in Between: Storytelling in Geriatric Medicine	UK	Non-clinician	Yes	Newsletter	Post-graduate	Undergraduate	Sharing experiences		
Hanna, M., Fins, J.J.	Acad Med	10.1097/00001888-200603000-00016.	2006	Viewpoint: power and communication: why simulation training ought to be complemented by experiential and humanist learning	USA	Clinician	No	Journal Article	Undergraduate		Teaching	Sharing experiences	
Campbell, J.	N.A.	none	1949	The hero with a thousand faces	USA	Non-clinician	No	Book	NA		Reflections		
Kathy	Healthcare Success	none		Healthcare Storytelling: The Best Marketing Magic and How to Do It	USA	Clinician	No	Online Article	Post-graduate		Sharing experiences		
Cabrera, D.	ICE Blog	none	2016	From Odysseus to Yoda. Storytelling in medical education	USA	Non-clinician	Yes	Blog	Undergraduate	Post-graduate	Teaching	Reflections	
Frank, A.W.	none	none	2013	The Wounded Storyteller: Body, Illness, and Ethics	USA	Non-clinician	Yes	Book	NA		Sharing experiences	Reflections	
Gu, Y.	Neohelicon	10.1007/s11059-018-0459-4	2018	Narrative, life writing, and healing: the therapeutic functions of storytelling	Hungary	clinician	Conflated with Narrative Medicine	Journal Article	Post-graduate		Sharing experiences	Reflections	teaching
Rivkin, S.	PharmExec	none	2021	Scientific Storytelling for Medical Affairs	USA	Clinician	Yes	Perspectives	Post-graduate		Sharing experiences		
Sandars, J.	Medical Teacher	10.1080/01421590903050374	2009	The use of reflection in medical education	UK	Clinician	Yes	Journal Article	Undergraduate	Post-graduate	Reflections	Teaching	Sharing experiences
Lehmann, S.	CLOSLER	none	2018	Storytelling in Medicine	USA	Clinician	Yes	Blog	Undergraduate	Post-graduate	Teaching	Reflections	Sharing experiences

Author	Journal	DOI	Year of Publication	Title	Country	Population	Definition of story telling	Document type	Undergraduate/ Graduate	Undergraduate/ Graduate	Purpose (Primary)	Purpose (Secondary)	Purpose (Tertiary)
Carmack, H. J.	Health Commun	10.1080/10410236.2010.484876	2010	Bearing witness to the ethics of practice: storying physicians' medical mistake narratives	USA	Clinician	No	Journal Article	Post-graduate		Reflections		
Paton, A.<br>Kotzee, B.	Health (London)	10.1177/1363459319889102	2021	The fundamental role of storytelling and practical wisdom in facilitating the ethics education of junior doctors	UK	Clinician	Yes	Journal Article	Post-graduate		Ethics	Teaching	
Chang, M.K., Lim, M.X., Tay, K., Lee, R.J., Sim, D.SW., Menon, S., Kanesvaran, R., Puvanendran, R., Krishna, L.K.R.	Asian Bioethics Review	10.1007/s41649-017-0003-2	2017	The Influence of Evolving Confucian Beliefs in the Selection of Proxy Decision-Makers at the End of Life in Singapore	Singapore	Non-clinician	No	Journal Article	NA		Ethics		
Krishna, L.	Ethics & Medicine	none	2013	Palliative care imperative: A framework for holistic and inclusive palliative care	Singapore	Clinician	No	Journal Article	Post-graduate		Ethics		
Krishna, L.	Ethics & Medicine	none	2011	The position of the family of palliative care patients within the decision-making process at the end of life in Singapore	Singapore	Non-clinician	No	Journal Article	NA		Ethics	Sharing experiences	
Krishna, L.K.R.<br>Alsuwaigh, R.<br>Miti, P.T.<br>Wei, S.S.<br>Ling, K.H.<br>Manoharan, D.	Am J Hosp Palliat Care	10.1177/1049909113500136	2014	The Influence of the Family in Conceptions of Personhood in the Palliative Care Setting in Singapore and Its Influence Upon Decision Making	Singapore	Non-clinician	No	Journal Article	NA		Reflections		
Ho, Z.J.M.<br>Krishna, L.K.R.<br>Goh, C.<br>Yee, C.P.A.	Palliat Support Care	10.1017/S1478951512000429	2012	The physician-patient relationship in treatment decision making at the end of life: A pilot study of cancer patients in a Southeast Asian society	Singapore	Non-clinician	No	Journal Article	NA		Sharing experiences		

Undergraduate	52	26%
Post-graduate	106	53%
NA	41	21%
**Total**	**199**	**100%**

**Purpose**		**Percentage %**
Sharing experiences	62	31
Reflections	47	24
Teaching	52	26
Public Education	12	6
Ethics	23	12
Physical and Psychological Health	3	2
**Total**	**199**	100

	**Countries**		**Percentage %**
	USA	137	68.5
	UK	15	7.5
	Canada	11	5.5
	Australia	8	4
	Brazil	1	0.5
**Asia**		**11**	**5.5**
	China	1	0.5
	Hong Kong	1	0.5
	India	1	0.5
	Japan	1	0.5
	Nepal	1	0.5
	Singapore	6	3
**Europe**		**11**	**5.5**
	Belgium	1	0.5
	Hungary	1	0.5
	Italy	3	1.5
	Spain	1	0.5
	Finland	1	0.5
	Netherlands	1	0.5
	Poland	2	1
	Sweden	1	0.5
**Middle East**		**6**	**3**
	Iran	2	1
	Egypt	1	0.5
	UAE	1	0.5
	Israel	2	1
**Total**		**200**	**100**
